# The fiber metabolite butyrate reduces gp130 by targeting TRAF5 in colorectal cancer cells

**DOI:** 10.1186/s12935-020-01305-9

**Published:** 2020-06-03

**Authors:** Yin Yuan, Bo Li, Yanbin Kuang, Shuo Ni, Aoxiang Zhuge, Jing Yang, Longxian Lv, Silan Gu, Ren Yan, Yating Li, Kaicen Wang, Liya Yang, Xueling Zhu, Jingjing Wu, Xiaoyuan Bian, Lanjuan Li

**Affiliations:** 1 State Key Laboratory for Diagnosis and Treatment of Infectious Diseases, The First Affiliated Hospital, School of Medicine, Zhejiang University, Hangzhou, 310003 China; 2 Collaborative Innovation Center for Diagnosis and Treatment of Infectious Diseases, Hangzhou, China; 3 Department of Respiratory Medicine, School of Medicine, Ren Ji Hospital, Shanghai Jiao Tong University, Shanghai, China; 4 Department of Orthopedics, Shanghai Pudong Hospital, Fudan University Pudong Medical Center, Shanghai, China

**Keywords:** Colorectal cancer, Sodium butyrate, GP130, TRAF5, Protein stability

## Abstract

**Background:**

Dietary fiber is effective for colorectal cancer (CRC) treatment. Interleukin-6 (IL-6) and its adaptors are potential targets for CRC therapy. Butyrate, a metabolite of dietary fiber, is a new, highly safe type of targeted drug.

**Methods:**

In this study, Cell Counting Kit-8 cell viability and wound healing assays, western blot analysis, immunofluorescence staining, and xenograft tumor mouse models were used to evaluate the anticancer effect of butyrate and its possible mechanism in vivo and in vitro.

**Results:**

Dietary fiber and sodium butyrate (NaB) decreased CRC burden by decreasing IL-6 receptor gp130 and blocking IL-6/JAK2/STAT3 axis activation in vitro and in vivo. Furthermore, NaB reduced the gp130 protein level by regulating its degradation rate via targeting TRAF5.

**Conclusions:**

The fiber metabolite butyrate inhibits CRC development by reducing gp130 via TRAF5.

## Background

Colorectal cancer (CRC) is among the most common cancers worldwide [1–3]. Genetic factors, dietary habits, lifestyle, and inflammation are related to the occurrence of CRC [4–7]. In recent years, the morbidity of CRC has continued to rise, and the age of CRC patients has tended to be younger because of changes in people’s dietary habits and dietary structure [8, 9]. Although the five-year survival rate of CRC patients has increased significantly because of the extensive application of screening, surgery, chemoradiotherapy, and other treatment methods, the sequelae of surgical treatment and side effects caused by chemoradiotherapy render the prognosis of CRC unsatisfactory [10, 11]. Therefore, more efficient and safer therapeutic approaches for CRC are needed.

Accumulating evidence indicates that dietary adjustment is an important approach to prevent and treat CRC [12–14]. A study by Song et al. found that the consumption of a large amount of dietary fiber can reduce the mortality of CRC patients and confer extra benefits to them [15]. In addition, gut microbiota was also involved in this process [16]. After entering the intestinal tract, the gut microbiota decompose dietary fiber into short-chain fatty acids (SCFAs), including butyric acid, a short-chain fatty acid [17, 18]. Butyric acid can inhibit inflammatory reactions and apoptosis and also regulate the gut microecology [19]. O’Keefe et al. reported that CRC patients lacking butyrate-producing *Clostridium* species in the gut microbiota tend to have poor prognoses, suggesting that butyric acid may be related to the prognosis of CRC [20]. In addition, several studies have shown that butyrate can induce the apoptosis and differentiation and inhibit the proliferation of CRC cells. The proposed mechanisms include the functions of butyrate as a histone deacetylase (HDAC) inhibitor and DNA methylation inhibitor, among others [21–23]. However, the mechanism by which butyrate prevents and treats CRC has not yet been fully elucidated.

Dysregulation of several key signaling molecules is related to the occurrence and development of CRC, especially Interleukin-6 (IL-6) [24, 25]. IL-6 is a multifunctional cytokine, and its dysfunction and abnormal expression often lead to disease [26, 27]. Kim et al. revealed that serum IL-6 levels were significantly increased in CRC patients and that serum IL-6 levels were positively correlated with the mortality and prognosis of CRC [28]. IL-6 exerts its biological effects by binding to its receptors, the IL-6 α receptor (glycoprotein 80, gp80), and the IL-6 β receptor (glycoprotein 130, gp130) [29]. A homodimer composed of IL-6 and gp130 can phosphorylate downstream Janus tyrosine kinases (JAKs), which then activate various downstream transcription factors [30]. The IL-6/JAK2/STAT3 pathway was discovered to be constitutively activated in human CRC and significantly related to cancer cell proliferation, invasion, and migration [31, 32]. Grivennikov et al. found that in CRC mouse models, IL-6 promoted the occurrence of CRC, and genetic knockout of IL-6 or STAT3 suppressed the occurrence of CRC [33]. Therefore, blocking the IL-6/JAK2/STAT3 signaling axis and its biological effects may be a therapeutic strategy for CRC.

Tumor necrosis factor receptor-associated factors (TRAFs) is an important kind of intracellular signaling protein, which is involved in the activation of a variety of signaling pathways and the proliferation and apoptosis of tumor cells [34, 35]. TRAF5 is a kind of TRAF that has been shown to be a negative regulator of gp130. Hiroyuki et al. revealed that TRAF5 could constitutively connect to gp130, occupying the binding sites of STAT3, inhibiting the dimerization of gp130, and thereby suppressing the activation of IL-6/JAK2/STAT3 signaling [36]. Therefore, we consider TRAF5 as a potential target for CRC therapy.

Our previous study has shown that dietary fiber metabolite butyrate can significantly inhibit the inflammatory response and the expression of IL-4, IL-6, IL-10, and other inflammatory factors in mouse models of nonalcoholic steatohepatitis [37]. Inflammation is closely associated with the initiation and development of CRC [38, 39]. Therefore, we speculated that butyrate may function as an anticancer drug by regulating inflammation-related signaling pathways. In this study, we first revealed the therapeutic effect of high-fiber diet in inhibiting the progression of CRC in xenograft tumor mouse models. Next, we identified butyrate as a major component for CRC treatment. Then, we revealed the role of butyrate in suppressing the development of human CRC cells via blocking activation of the IL-6/JAK2/STAT3 signaling pathway. Furthermore, we found that butyrate exhibited its function by up-regulating the TRAF5 level and enhancing the combination between TRAF5 and gp130, thereby reducing the IL-6 receptor gp130. Our results may provide a novel approach for molecular targeted therapy for CRC.

## Methods

### Cell lines and reagents

The HCT-116 and HT-29 human CRC cell lines were purchased from the ATCC (Manassas, VA, USA). RPMI 1640 medium (Gibco, Gaithersburg, MD, USA) supplemented with 10% fetal bovine serum (FBS) and 1% penicillin/streptomycin was used to culture both kinds of cells. Cells were cultured in a 37 °C incubator with 5% CO_2_. Sodium butyrate (NaB) was purchased from Aladdin (Shanghai, China). Recombinant human IL-6 protein was obtained from R&D Systems (Minneapolis, MN, USA).

### Cell viability assay

An enhanced Cell Counting Kit-8 (CCK-8; Beyotime, Shanghai, China) was used to measure cell viabilities. HCT-116 cell and HT-29 cell were cultured in 96-well plates with 0–10 mM NaB at a density of 5000 cells per well for 0–36 h. Then, CCK-8 assay solution was added to each sample. All cells were incubated for 1.5 h in the dark. The absorbance was assessed at 450 nm. For each condition, 6 independent biological duplicates were assessed.

### EdU cell proliferation assay

The cell proliferative ability was assessed using an EdU cell proliferation kit (Beyotime). According to the instructions, EdU working solution was added to cells pretreated with 5 mM NaB for 24 h. Then, all the samples were cultured in an incubator for 2 h and fixed for 15 min. All cells were stained with EdU and Hoechst 33342. The cells were counted and imaged with an Olympus FSX100 microscope (Olympus, Tokyo, Japan).

### Apoptosis assay

Apoptosis was assessed by fluorescence-activated cell sorting (FACS) analysis according to the instructions of the Annexin V-FITC detection kit (KeyGEN BioTECH, Nanjing, China). HCT-116 and HT-29 cells were treated with 5 mM NaB or 10 mM NaB for 24 h. Then, all the cells were collected and incubated with FITC-conjugated Annexin V and propidium iodide (PI) dye. Apoptosis was measured by flow cytometry (BD Biosciences Franklin Lakes, NJ, USA). Results were analyzed with CellQuest software.

### Colony formation assay

Cells in logarithmic phase were collected and resuspended in RPMI 1640 medium containing 5 mM NaB. Then, the cells were transferred to 6-well plates (400 cells per well) and incubated for 14 days. All media were replaced daily. Then, colonies were fixed for 15 min and stained with 1% Giemsa stain solution (Solarbio, Beijing, China) for 30 min. All colonies were counted.

### Wound healing assay

HCT-116 cell and HT-29 cell were cultured in 12-well plates. At 100% confluence, scratches of equal widths were created in the cell monolayers with sterile 10-µl pipette tips. After washing with PBS, all cells were cultured in FBS-free RPMI 1640 medium containing 5 mM NaB for 24 h. Images were acquired with an Olympus FSX100 microscope (Olympus). The wound closure percentage was calculated with ImageJ software (USA).

### Transwell assay

Transwell chambers (Corning Costar, Cambridge, MA, USA) with 8-µm pores were used for this assay. For the cell migration assay, cells were cultured in FBS-free RPMI 1640 medium overnight and transferred to the upper chambers (10^5^ cells per well) the next day. Then, medium with 10% FBS and 5 mM NaB was added to the lower chambers. Twenty-four hours later, the filter membranes were fixed for 15 min after washing with PBS and stained with 1% Giemsa stain solution for 30 min. Then, the cells remaining on the upper surface of the membranes (non-migrated cells) were gently removed with cotton swabs. The cells remaining on the lower surface (migrated cells) were counted and imaged. For the cell invasion assay, filter membranes precoated with a 1:6 dilution of Matrigel (BD Bioscience, San Diego, CA, USA) were used instead of uncoated filter membranes. The other steps were performed as described for the cell migration assay.

### Western blot analysis

Cells were collected for protein extraction after treatment with 5 mM NaB for 24 h followed by 25 ng/ml IL-6 for 30 min. RIPA lysis buffer containing protease and phosphatase inhibitors was used to extract cellular proteins. Proteins were separated by SDS-PAGE and then transferred to PVDF membranes and probed with specific primary antibodies and secondary antibodies. An enhanced chemiluminescence detection kit (Beyotime) was used to visualize the bands. Antibodies specific for gp80 (sc-373780, 1:100) and gp130 (sc-376280, 1:100) were purchased from Santa Cruz Biotechnology (Dallas, TX, USA). Antibodies specific for STAT3 (#9139, 1:1000), JAK2 (#3230, 1:1000), p-STAT3 (#9145, 1:1000), p-JAK2 (#3771, 1:1000), and TRAF5 (#41658, 1:1000) were obtained from CST (Beverly, MA, USA). The anti-GAPDH antibody (BM1623, 1:1000), anti-β-actin antibody (BM0627, 1:1000), anti-α-tubulin antibody (BM1452, 1:1000), anti-rabbit IgG-HRP antibody (BA1054, 1:5000), and anti-mouse IgG-HRP antibody (BA1050, 1:5000) were purchased from Boster Biological Technology (Wuhan, China).

### Protein stability assay

Cells were pretreated with 5 mM NaB for 24 h. Then, 0.5 h, 1 h, and 2 h before the cells were collected, 30 µg/ml cycloheximide (CHX; MCE, Monmouth Junction, NJ, USA) was added to all cells to block protein translation. The gp130 protein level was assessed using the western blot assay.

### TCGA database and analysis

TRAF5 genes were analyzed by GEPIA web tools (http://gepia.cancer-pku.cn/) based on the TCGA database.

### SCFA assay

SCFAs were assessed from fecal samples homogenized in buffers. The suspension was centrifuged, and the supernatant was collected for analysis. A gas chromatography and a fused silica capillary column (Nukon™, Bellefonte, PA, USA) were used for the assay.

### Cell transfection

HT-29 cells were transfected with siRNA using Lipofectamine 3000 (Invitrogen, Carlsbad, CA, USA) according to the instructions. Briefly, cells were seeded in 6-well plates at a density of 2 × 10^4^ cells per well the day before transfection. The cells were then transfected with 20 nM siRNA the next day. All media were replaced with culture medium 6 h after transfection, and the transfection efficiency was assessed after 48 h using a fluorescence microscope. The transfected HT-29 cells were then used for experiments according to protocols. The TRAF5 siRNA kit was purchased from Ribobio (Guangzhou, China).

### Immunoprecipitation

For this assay, all cells were lysed in NP-40 buffers and centrifuged at 12,000 rpm for 10 min. Proteins were immunoprecipitated from supernatants with primary antibodies immobilized on protein G agarose beads overnight at 4 °C. Then, all proteins were collected for western blot analysis.

### RNA extraction and qRT-PCR

Cells treated with 5 mM NaB for 24 h and 25 ng/ml IL-6 for 30 min were collected for RNA extraction. RNA was extracted with an RNeasy Mini Kit (Qiagen, Valencia, USA) and then reverse-transcribed into cDNA. The mRNA expression levels were assessed by a VII A7 real-time PCR system (Applied Biosystems, Foster, CA, USA). The primers are listed in Additional file 1: Table S1.

### Immunofluorescence

All cells were treated with 5 mM NaB for 24 h and 25 ng/ml IL-6 for 30 min, fixed for 15 min, and blocked with 5% goat serum for 1 h. Then, the cells were incubated with anti-p-STAT3 antibody (CST, 1:200) overnight at 4 °C and with a Cy3-conjugated secondary antibody (Beyotime, 1:500) for 1 h the next day. Images were acquired with a Zeiss LSM T-PMT confocal microscope (Zeiss, Jena, Germany).

### Mouse xenograft tumor model

All animal experiments were performed according to guidelines approved by the Animal Care Committee of Zhejiang University School of Medicine. A total of 10^6^ HT-29 cells resuspended in 200 µl FBS-free RPMI 1640 medium were injected subcutaneously into BALB/c athymic nude mice (6-week-old, female). Tumor volumes were measured every 2 days. When the tumor volume reached 60–80 mm^3^, the mice were divided into different groups randomly. For the dietary fiber experiments, the low-fiber diet (LFD) group was fed with low-fiber food (20% fiber) and the high-fiber diet (HFD) group was fed with a high-fiber food (70% fiber) for 2 weeks. All diets were purchased from Test Diet (Richmond, IN). After 2 weeks, all animals were sacrificed, and tumor tissues and normal peritumoral tissues were collected. All tissues were frozen in liquid nitrogen and fixed with 4% paraformaldehyde for western blot analysis, hematoxylin and eosin (HE) staining, and immunofluorescence staining. For butyrate experiments, the control group was intraperitoneally injected with normal saline every day, while the NaB group was intraperitoneally injected with 200 mg/kg NaB. Other steps were performed as described above.

### Statistical analysis

Data were analyzed using SPSS software (Chicago, IL, USA) and GraphPad Prism 7.0 (San Diego, CA, USA). The differences between groups were calculated by ANOVA and the Student’s t-test. All data are presented as the mean ± SEM. A p < 0.05 was considered significant.

## Results

### High-fiber diet inhibits CRC development by producing butyrate in xenograft tumor models

**Fig. 1:**
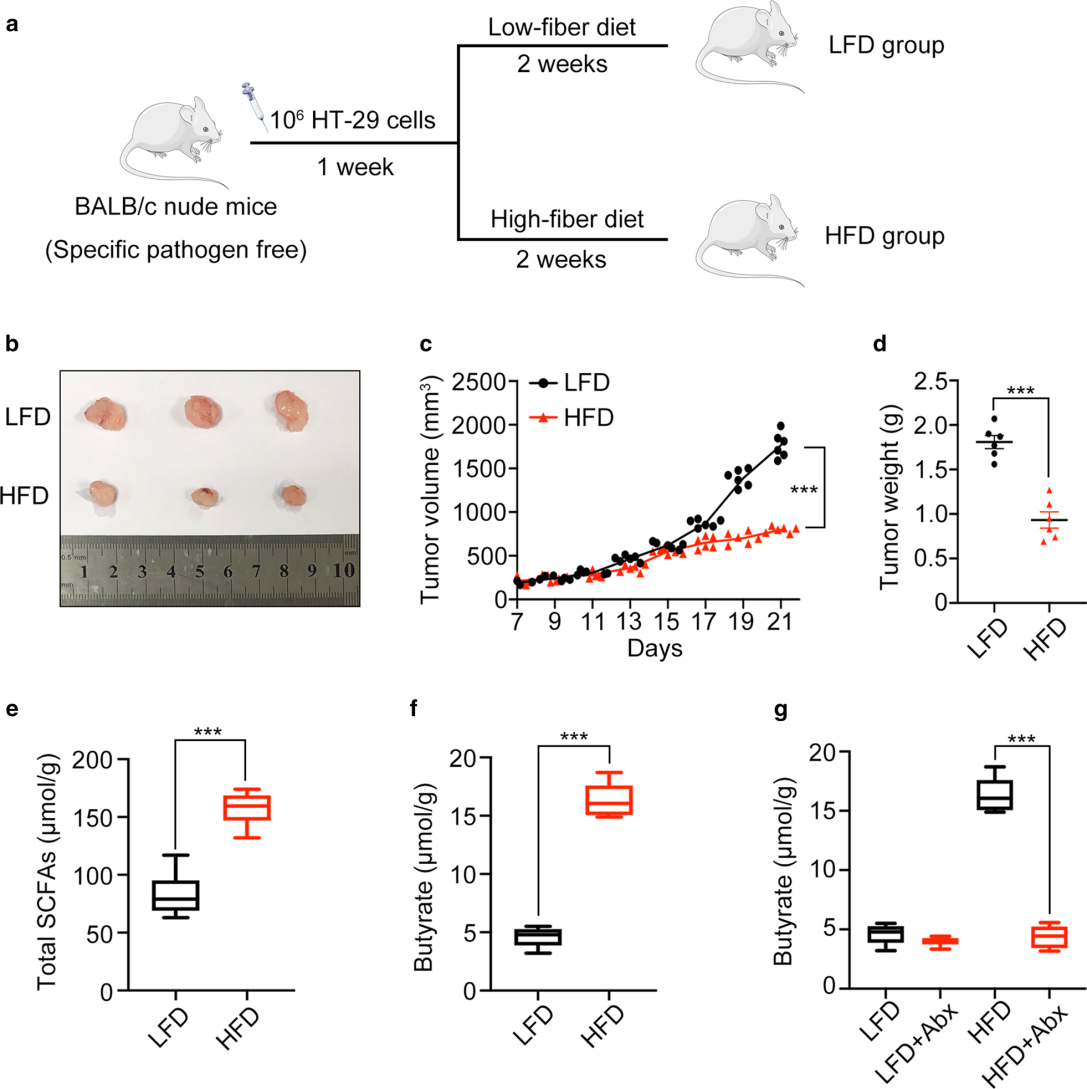
High-fiber diet (HFD) inhibited CRC development. **a** Protocols of the dietary fiber experiments. **b**–**d** Images, volumes, and weights of tumors. **e**, **f** Concentration of total SCFAs and butyrate in fecal samples. **g** Concentration of fecal butyrate in mice treated with or without antibiotics (Abx). (***p < 0.001)

To investigate whether dietary fiber could inhibit tumor development in vivo, a low-fiber diet (LFD) and high-fiber diet (HFD) were used to treat xenograft tumor mice (Fig. 1a). Both tumor volume and tumor weight were lower in the HFD group than the LFD group (p < 0.001) (Fig. 1b–d). These results showed that the development of CRC was inhibited by dietary fiber. SCFAs are metabolites of fiber, so we measured the level of SCFAs in feces. In comparison to the LFD group, the fecal level of total SCFAs increased in the HFD group (p < 0.001) (Fig. 1e). Furthermore, we observed a significant increase in fecal butyrate (a main type of SCFAs) in the HFD group (p < 0.001) (Fig. 1f). To further explore the impact of the gut microbiota in fecal butyrate, we administered Abx to SPF mice to deplete their gut flora. Under the condition of HFD feeding, the fecal butyrate level of Abx-treated mice decreased significantly (p < 0.001) (Fig. 1g). Together, these results showed that dietary fiber could inhibit the development of CRC and that the gut microbiota was involved in metabolizing the fiber into butyrate.

### NaB reduces tumor burden and inhibits JAK2/STAT3 signaling axis activation in vivo

**Fig. 2:**
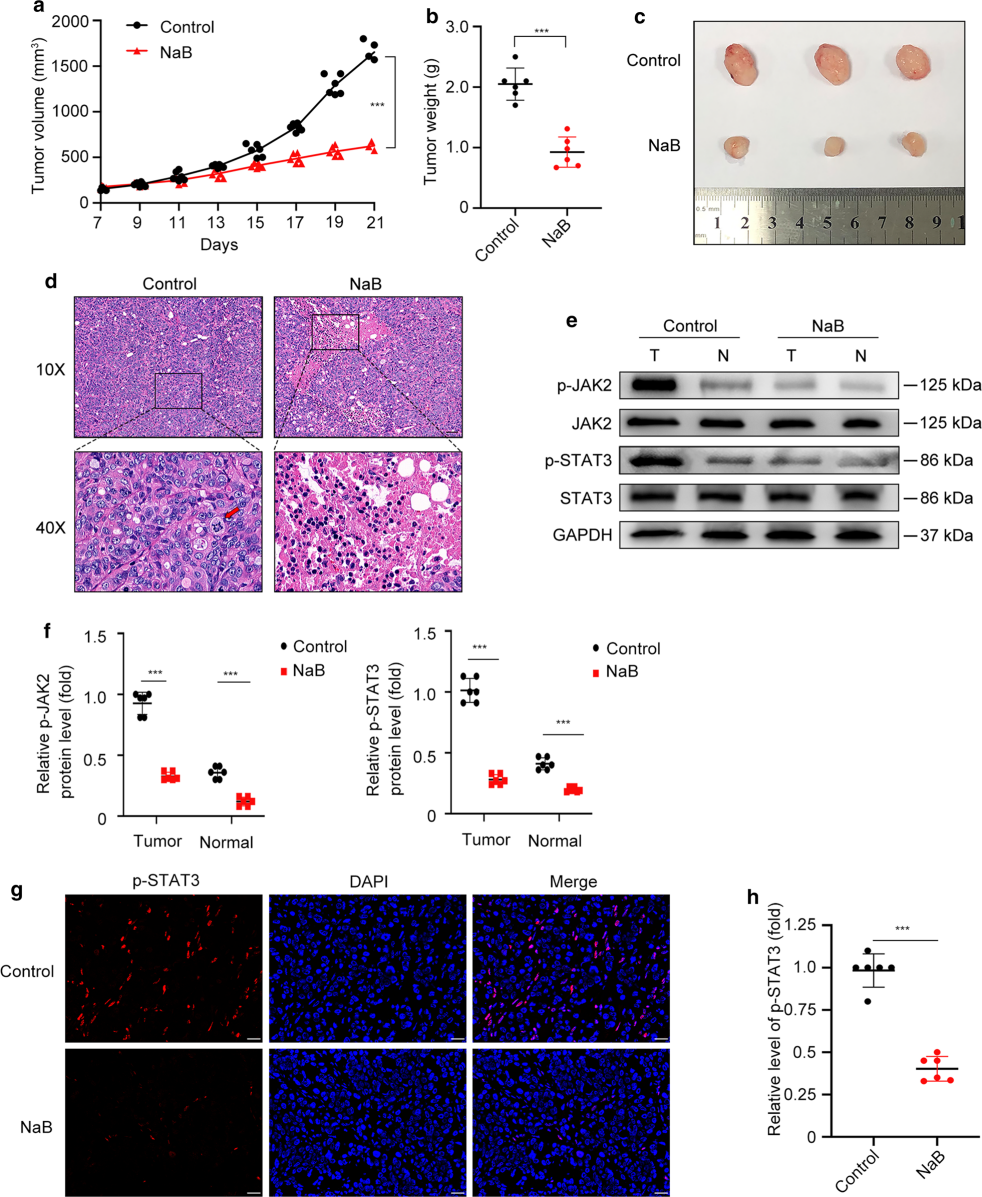
Sodium butyrate (NaB) reduced HT-29 xenograft tumor burden in vivo. **a**–**c** Images, volumes, and weights of tumors. **d** HE staining of tumor tissues. The arrow indicates pathological mitosis. Scale bar represents 50 μm. **e**, **f** Western blotting of p-JAK2 and p-STAT3 protein levels in tumor tissues (T) and normal peritumoral tissues (N) from mice. **g**, **h** Immunofluorescence staining specific for p-STAT3 proteins in mouse tumor tissues. Scale bar represents 20 μm. (***p < 0.001)

To demonstrate whether NaB can inhibit tumor growth in vivo, we established nude mouse xenograft tumor models. The volume and weight of tumors in NaB group mice were significantly lower compared with control group mice (p < 0.001) (Fig. 2a–c). The HE staining results showed a high level of pathological mitosis (arrow in Fig. 2d) but no necrosis in tumor tissues from the control group. However, the tumor tissues from the NaB group exhibited a large number of necrotic areas (Fig. 2d). Activation of the JAK2/STAT3 signaling axis was found to be closely related to the development of CRC [31]. The western blotting results showed that protein levels of p-JAK2 and p-STAT3 decreased significantly in NaB group in both tumor (T) and normal peritumoral (N) tissues (p < 0.001) (Fig. 2e, f). Immunofluorescence staining of tumor tissues revealed similar results. The protein levels of p-STAT3 protein were significantly lower in tumor tissues from the NaB group than the control group (Fig. 2g, h). Collectively, NaB could reduce tumor burden and inhibit JAK2/STAT3 signaling axis activation in mouse xenograft tumor models.

### NaB inhibits CRC cell proliferation in vitro

**Fig. 3:**
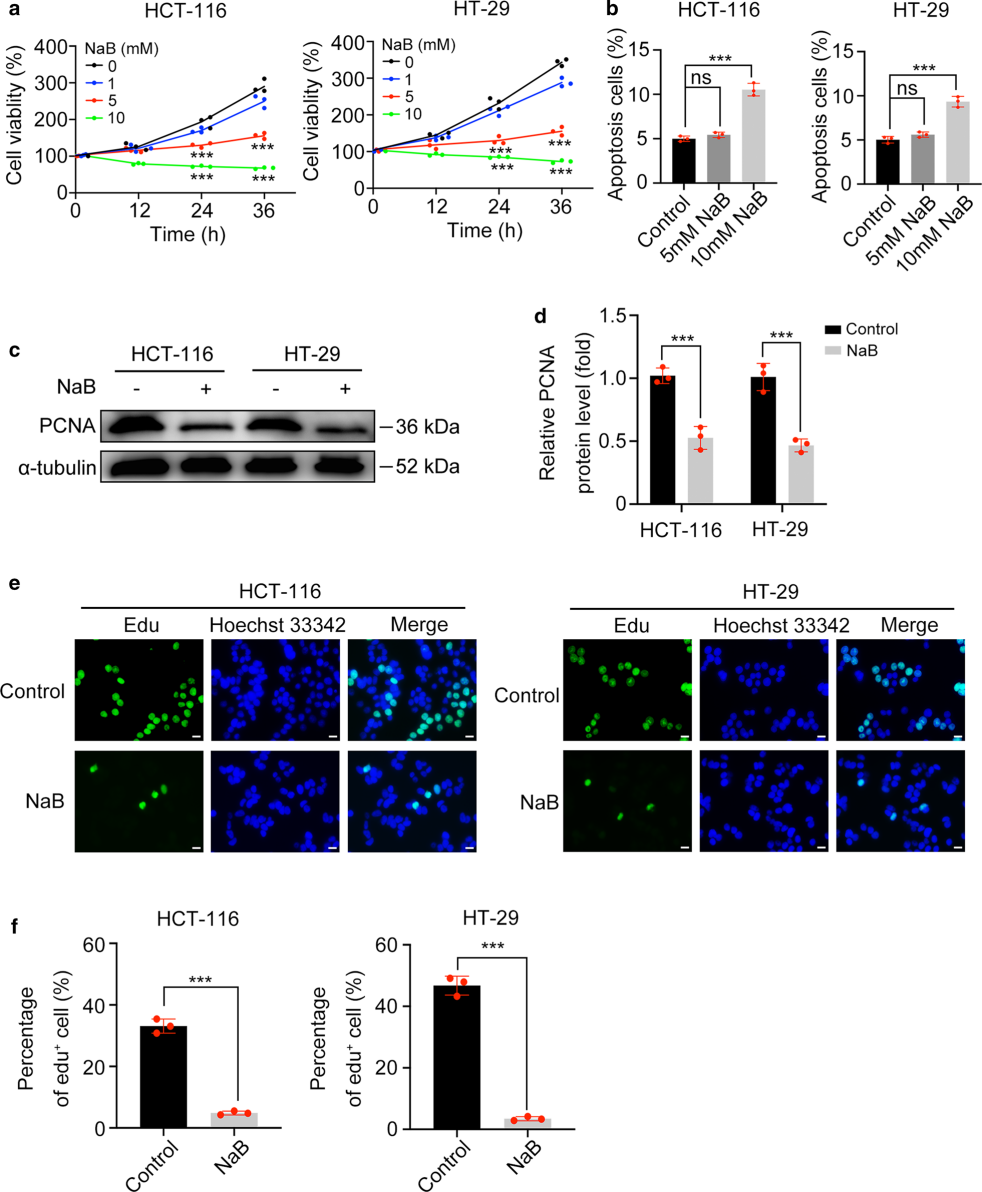
NaB inhibited proliferation of HCT-116 and HT-29 cells. **a** Cell viabilities of HCT-116 and HT-29 cells treated with 0–10 mM NaB for 0–36 h. **b** Cell apoptosis of HCT-116 and HT-29 cells treated with 5 and 10 mM NaB for 24 h. **c**, **d** Western blot analysis of PCNA protein levels in HCT-116 and HT-29 cells treated with 5 mM NaB for 24 h. **e**–**f** Images and percentages of EdU-positive cells. Scale bar represents 10 μm. (***p < 0.001; *ns* not significant)

Cell proliferative abilities were assessed using several different approaches. First, CCK-8 assays were used to measure cell viabilities of HCT-116 and HT-29 cells. Cell viabilities decreased significantly upon treatment of 5 mM and 10 mM NaB (p < 0.001), but not significantly upon 1 mM NaB treatment (p > 0.05) (Fig. 3a). Next, we measured cell apoptosis by FACS analysis. Neither cell line exhibited significant apoptosis when treated with 5 mM NaB (p > 0.05), but they did with 10 mM NaB treatment (p < 0.001) (Fig. 3b). Therefore, we considered 5 mM NaB as the optimal concentration to inhibit proliferation of CRC cells because of the lower levels of cytotoxicity in vitro. In addition, pretreatment with other kinds of SCFAs (acetate, propionate, and valerate, 0.1 mM) did not significantly reduce the cell viability (Additional file 2: Figure S1). Proliferating cell nuclear antigen (PCNA) is a common indicator of cell proliferation. NaB treatment decreased the PCNA protein level (p < 0.001) (Fig. 3c, d), suggesting that the cell proliferation was inhibited by NaB. The results of the EdU cell proliferation assay showed that the EdU-positive cell percentage also decreased in cells treated with NaB (p < 0.001) (Fig. 3E-F). Collectively, these results showed that the cell proliferative ability could be inhibited by NaB.

### NaB inhibits the colony formation, migration, and invasion ability of CRC cells

**Fig. 4:**
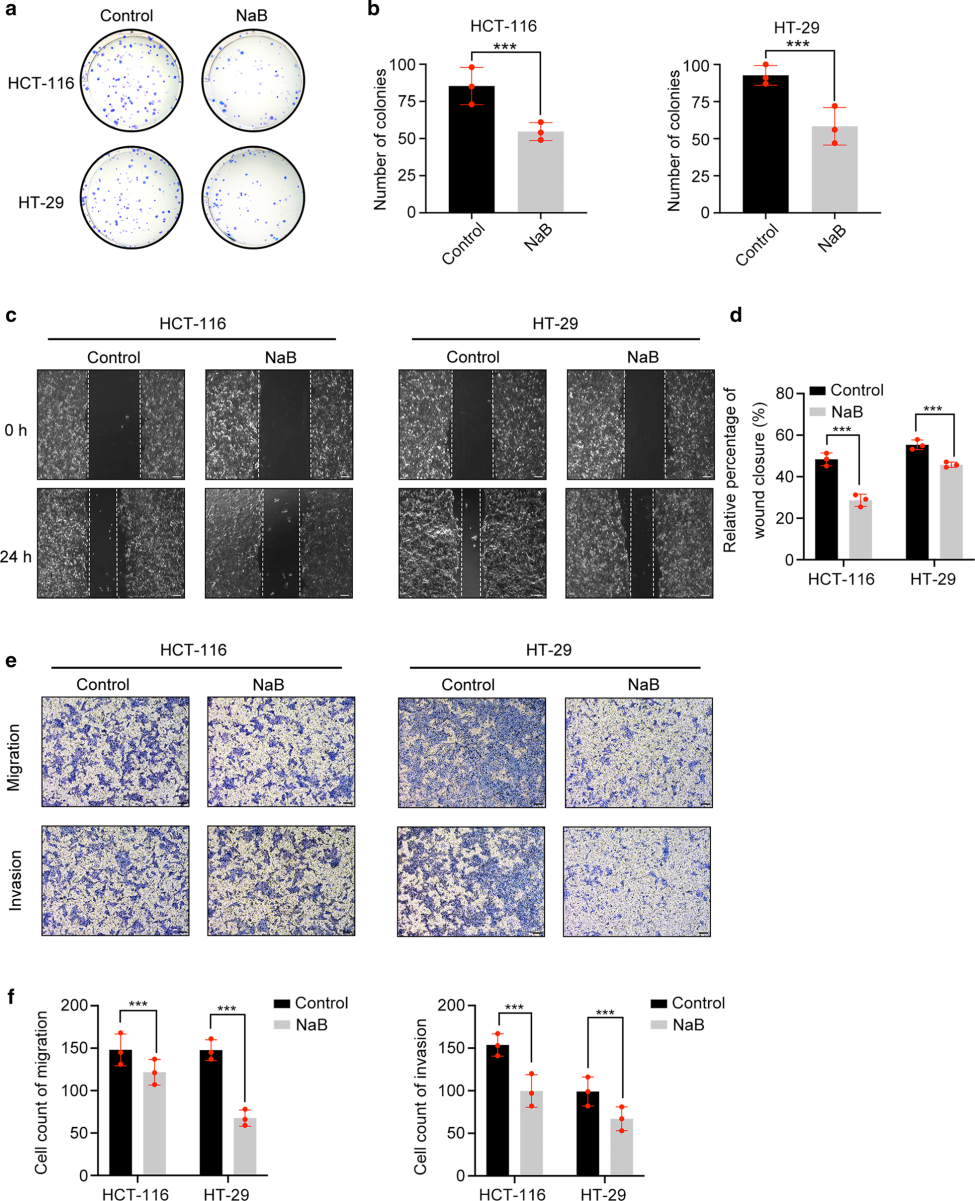
NaB inhibited cell colony formation and translocation. **a**, **b** Images and amounts of HCT-116 and HT-29 cell colonies treated with 5 mM NaB for 2 weeks. **c**, **d** Wound healing percentages of HCT-116 and HT-29 cells treated with 5 mM NaB for 24 h. Scale bar represents 100 μm. **e**, **f** Images and counts of migrated and invaded cells assessed by the Transwell assay. Scale bar represents 100 μm. (***p < 0.001)

Colony formation ability was evaluated using a colony formation assay. The colony formation ability of both kinds of cells decreased with NaB treatment (p < 0.001) (Fig. 4A-B). The migration of CRC cells was evaluated by wound healing assays. Wound closure rates decreased after NaB treatment (p < 0.001) (Fig. 4C-D). In addition, a Transwell experiment was used to assess the migration and invasion abilities. Consistent with the results in the wound healing assay, treatment with NaB could inhibit the migration of both cell lines in the Transwell assay (p < 0.001). Similar results were observed in the cell invasion assay. Cell invasion abilities decreased after treatment with NaB (p < 0.001) (Fig. 4e, f). These results showed that NaB could reduce the colony formation, migration, and invasion abilities of CRC cells.

### NaB inhibits IL-6/JAK2/STAT3 signaling activation

**Fig. 5:**
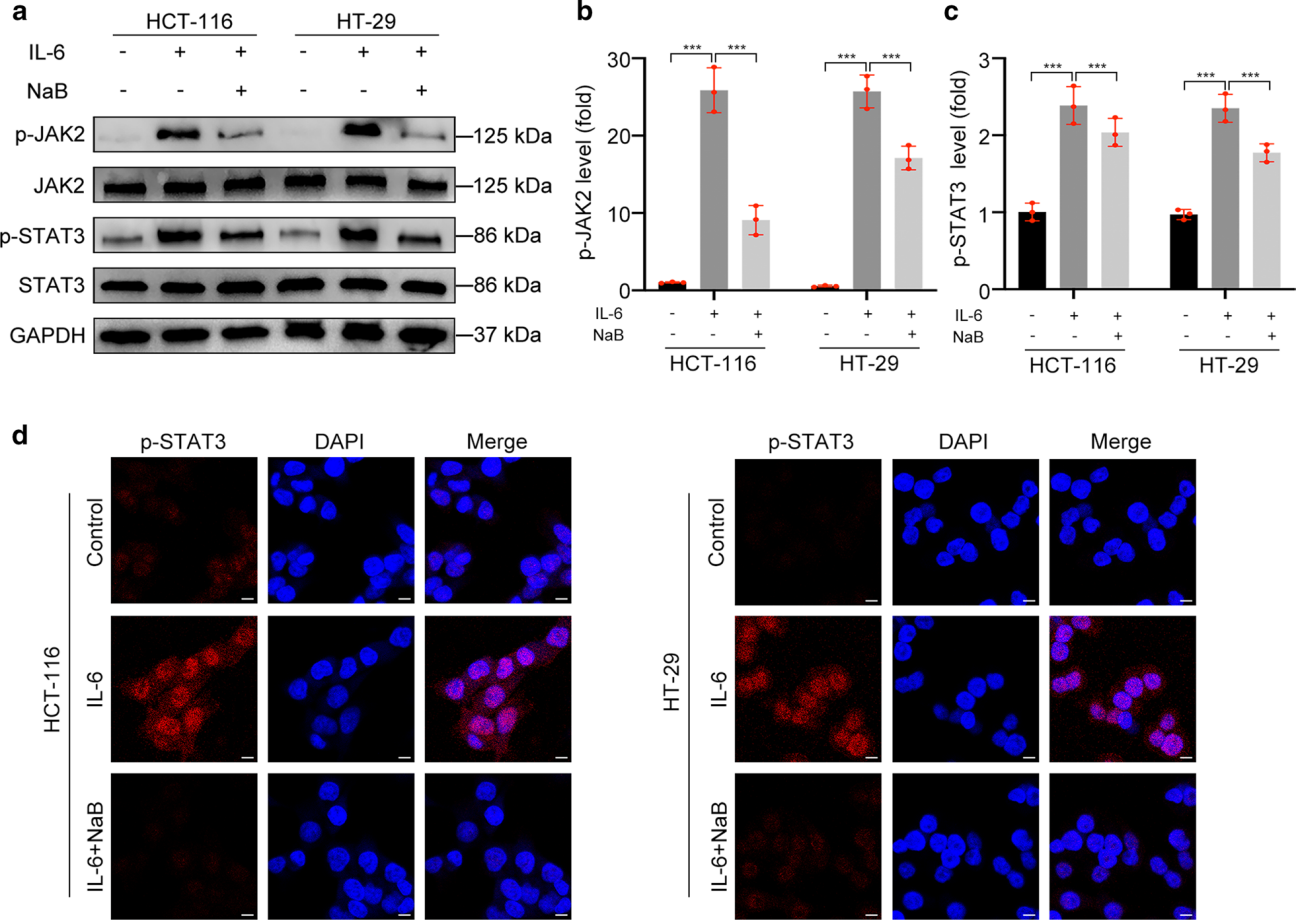
NaB inhibited activation of the IL-6/JAK2/STAT3 signaling pathway. **a**–**c** Western blot analysis of p-JAK2, JAK2, p-STAT3, and STAT3 protein levels in cells treated with NaB (5 mM) for 24 h and IL-6 (25 ng/ml) for 30 min. **d** Immunostaining for p-STAT3 (red) and DAPI (blue) in cells pretreated with or without NaB (5 mM) for 24 h and IL-6 (25 ng/ml) for 30 min. Scale bar represents 10 μm. (***p < 0.001)

We further explored the mechanism underlying the NaB effect. Decreases in p-JAK2 and p-STAT3 were found in vivo, indicating that NaB might exhibit its effect by targeting the JAK2/STST3 axis. However, JAK2/STAT3 signaling was not naturally activated in vitro because there no cytokine was detected in the RPMI-1640 medium. Thus, IL-6 was added to activate JAK2/STAT3 signaling. We first measured the total protein level and phosphorylation level of JAK2 and STAT3. The phosphorylation levels of JAK2 and STAT3 were significantly increased in cells stimulated with IL-6 (p < 0.001), while pretreatment with NaB for 24 h could significantly decrease these levels (p < 0.001) (Fig. 5a–c). We obtained similar results in the immunofluorescence assay. The protein level of p-STAT3 increased in cells stimulated with IL-6 and decreased in cells pretreated with NaB (Fig. 5d). These results indicated that NaB could inhibit IL-6/JAK2/STAT3 signaling activation.

### NaB reduces the protein level of gp130 by decreasing its protein stability

**Fig. 6:**
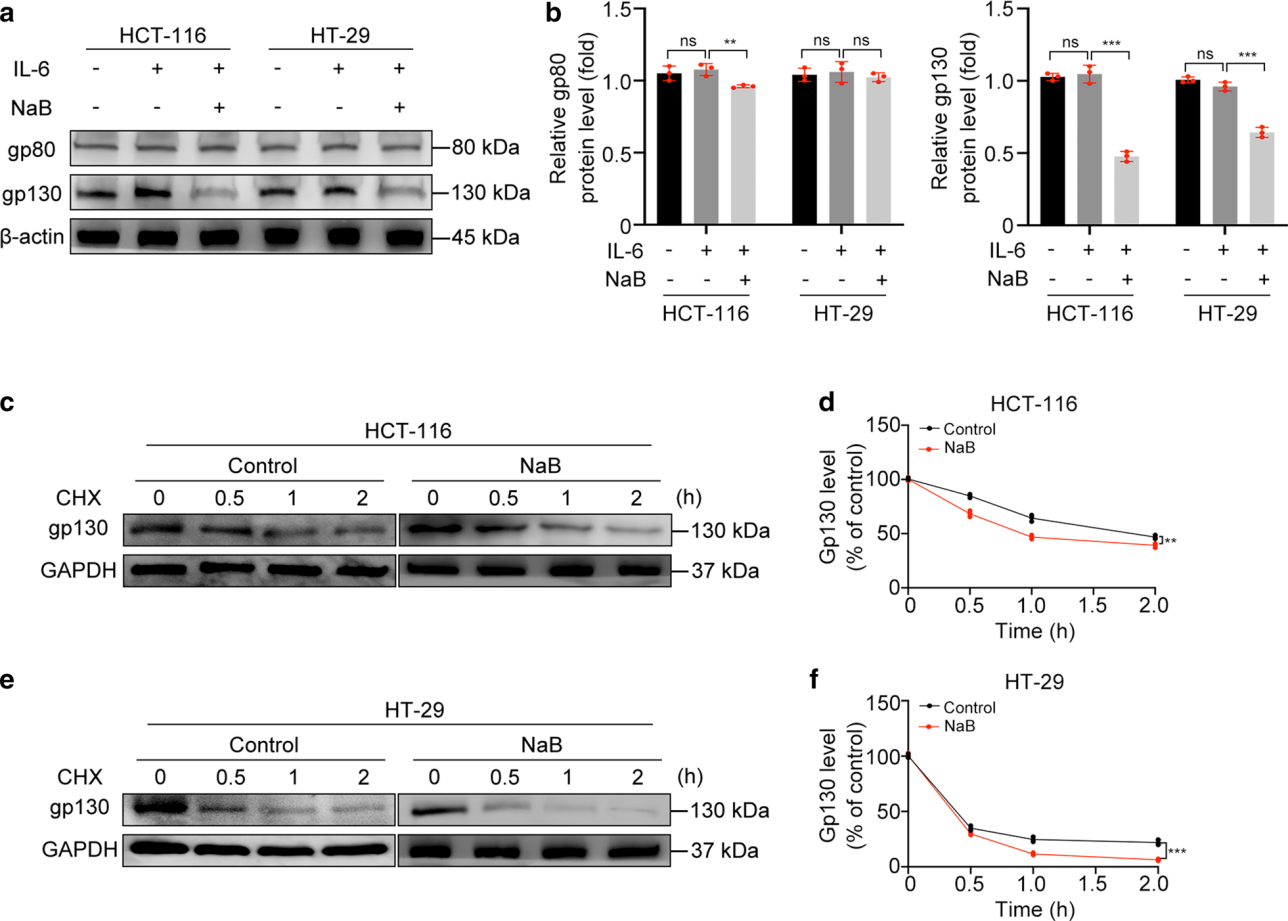
NaB decreased the IL-6 receptor gp130 level by accelerating its degradation. **a**, **b** Western blot analysis of gp80 and gp130 protein levels. **c**–**f** Western blotting of gp130 protein levels in cells treated with NaB (5 mM) for 24 h and CHX (30 µg/ml) for 0, 0.5, 1, and 2 h. (**p < 0.01; ***p < 0.001; *ns* not significant)

To further explore the mechanism by which NaB inhibits the hyperactivation of IL-6/JAK2/STAT3 signaling, we measured protein levels and gene expression levels of the IL-6 receptors gp80 and gp130. The western blotting results showed a decrease in the gp80 protein level in HCT-116 cells treated with NaB (p < 0.01), but no change in HT-29 cells (p > 0.05). However, the gp130 protein level in both groups of cells decreased significantly (p < 0.001) (Fig. 6a, b), which suggested that NaB might be a specific inhibitor of gp130. Interestingly, the qRT-PCR results showed no significant change in the expression level of either the gp80 or gp130 gene upon treatment of cells with either IL-6 or NaB (p > 0.05) (Additional file 3: Figure S2A, B). Therefore, we concluded that NaB could decrease the protein level of gp130 but could not inhibit its gene expression. Next, we measured the protein stability of gp130. After protein translation was blocked with the protein translation inhibitor CHX, all cells were collected at different time points for measurement of their gp130 protein level. The degradation rate of gp130 increased in both cell lines treated with NaB (p < 0.01) (Fig. 6c–f), indicating that NaB could decrease the protein stability of gp130. In conclusion, these results indicated that NaB could increase the protein degradation rate and reduce the protein stability of gp130.

### NaB reduces the gp130 protein level by increasing TRAF5

**Fig. 7:**
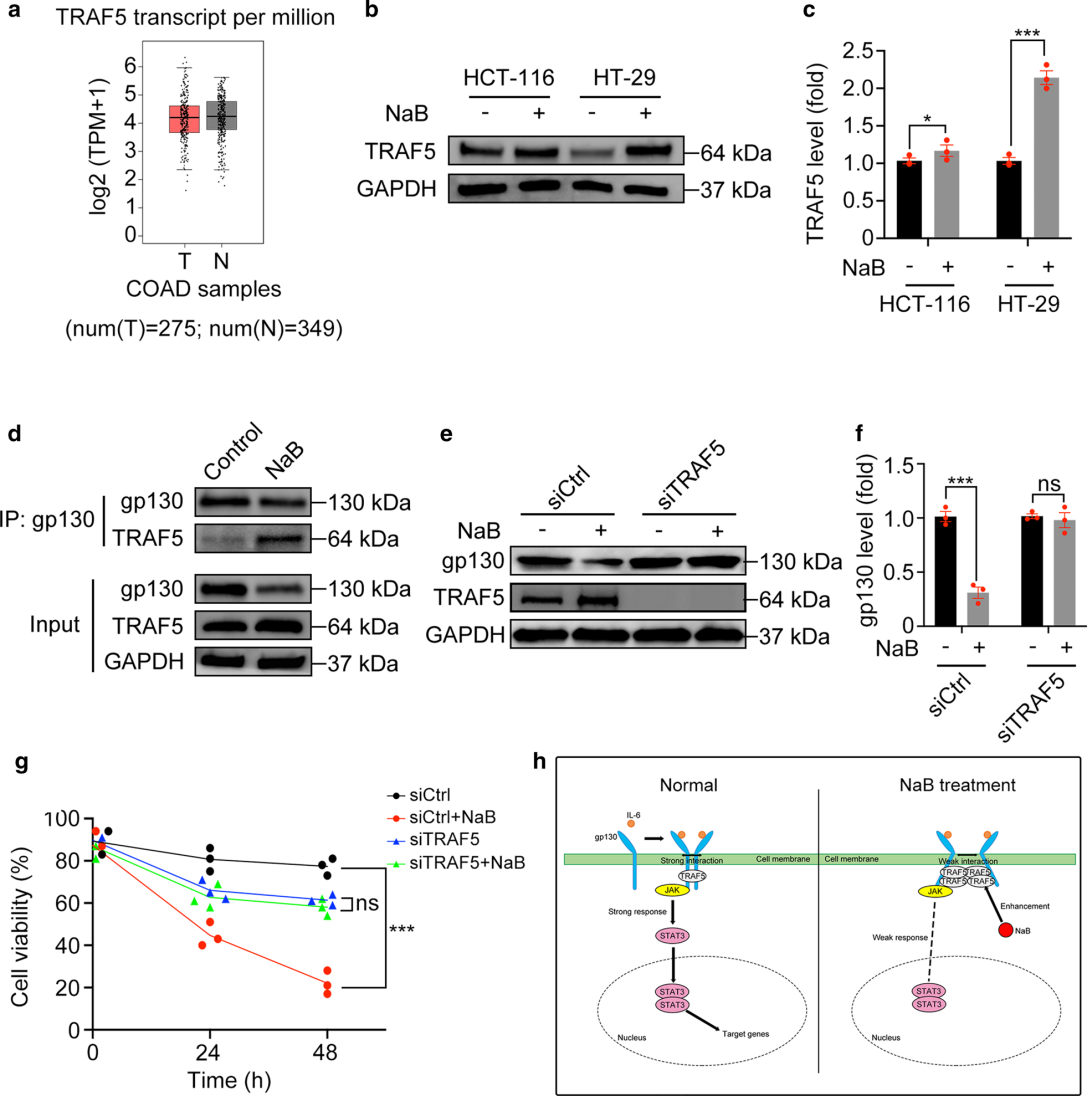
NaB accelerated gp130 degradation by increasing the level of TRAF5. **a** TRAF5 transcription analyzed by the GEPIA web tool. **b**, **c** Western blotting of TRAF5 protein levels in cells treated with NaB (5 mM) for 24 h. **d** Co-immunoprecipitation assay of gp130 and TRAF5 in HT-29 cells treated with 5 mM NaB for 24 h. **e**–**g** Rescue experiments of HT-29 cells transfected with TRAF5 siRNA. **h** A schematic diagram of the mechanism

The association of TRAF5 could weaken the dimerization of gp130, resulting in gp130 dysfunction and inactivation of IL-6/JAK2/STAT3 signaling [36]. According to GEPIA web tools based on the TCGA database (http://gepia.cancer-pku.cn/), in colon adenocarcinoma (COAD) patients, TRAF5 gene expression was lower in tumor tissues (T) than normal tissues (N) (Fig. 7a). First, we measured the TRAF5 protein level in both HCT-116 and HT-29 cells. NaB treatment increased the protein level of TRAF5 in both HCT-116 cells (p < 0.05) and HT-29 cells (p < 0.001) (Fig. 7b, c). Next, we detected the interaction of TRAF5 and gp130 using a co-immunoprecipitation assay. Notably, we found an association between TRAF5 and gp130, and this connection was partly enhanced by NaB treatment (Fig. 7d). Next, HT-29 cells were transfected with siRNA against TRAF5 to further explore the potential mechanism. Surprisingly, we found that NaB could not reduce the gp130 protein level and cell viability in cells treated with siTRAF5 (p > 0.05), but it could in cells treated with siCtrl (p < 0.001), indicating that TRAF5 knockdown could neutralize the effect of NaB (Fig. 7e–g). We hypothesized that by increasing the TRAF5 level and enhancing the association between traf5 and gp130, NaB could disable the biological function of gp130 and accelerate its degradation (Fig. 7h).

## Discussion

The main objective of this study was to explore the therapeutic effect and potential mechanism of butyrate as a novel inhibitor of gp130 in CRC. First, we found that a high-fiber diet could significantly inhibit CRC development in xenograft tumor mice. By assessing the substance in mice feces, we identified butyrate (a major metabolite of fiber) as a potential component for CRC therapy. In addition, we demonstrated that in specific pathogen-free (SPF) immunodeficient mice, the gut microbiota was necessary for metabolizing fiber into butyrate. Next, we revealed that butyrate significantly inhibited the proliferation, colony formation, and translocation abilities of HCT-116 and HT-29 cells. Butyrate exerted its function by reducing the gp130 level and thus blocking IL-6/JAK2/STAT3 signaling activation. However, butyrate did not significantly impact the gp130 gene expression level in either HCT-116 or HT-29 cells. Therefore, we speculated that butyrate might decrease the gp130 level by regulating the protein degradation and stability of gp130. Through a series of experiments, we confirmed that butyrate reduced the gp130 level by increasing the levels of the adaptor TRAF5 and enhancing the association of TRAF5 and gp130. As reported by Hiroyuki et al., the combination of TRAF5 and gp130 could inhibit the dimerization of gp130 and suppress the activation of JAK2/STAT3 signaling [36]. Based on the above findings, we speculated that butyrate could inhibit the dimerization of gp130 to accelerate its degradation by enhancing TRAF5. These results suggested that butyrate might be a drug useful for targeted CRC therapy.

CRC is a common malignancy related to genetic factors, diet, and inflammation [40, 41]. The consumption of large amounts of fiber results in a low risk of mortality [15]. In this study, we found that a high-fiber diet could significantly inhibit CRC development in xenograft tumor mice, in which butyrate was the main component. Moreover, we found that after depletion of the gut microbiota using antibiotics, the fecal butyrate level decreased significantly in the high-fiber diet mice. Thus, we concluded that the gut microbiota was necessary to metabolize the dietary fiber into butyrate.

We further explored the mechanism by which fiber can treat and prevent CRC. The dysregulation of key cytokines is important in the initiation and progression of CRC, particularly dysregulation of the IL-6 family [42, 43]. During the progression of cancer, hyperactivation of JAK2/STAT3 signaling results in a poor prognosis [44, 45]. In the present study, we confirmed that the major metabolite, butyrate, significantly inhibited the progression of CRC. Treatment with 5 mM butyrate did not induce significant apoptosis in vitro. Therefore, we considered 5 mM butyrate as the optimal and safer concentration for inhibiting CRC cell behaviors in vitro. Butyrate suppressed CRC by decreasing the gp130 level and inhibiting hyperactivation of IL-6/JAK2/STAT3 signaling. Furthermore, butyrate did not significantly impact gp80 gene expression or protein levels in either HCT-116 or HT-29 cells. Therefore, we speculated that butyrate might be a specific inhibitor of gp130 instead of gp80. Interestingly, the gp130 gene expression level did not change significantly in either HCT-116 or HT-29 cells upon treatment with butyrate, indicating that butyrate could not inhibit the gene expression of gp130. Therefore, we speculated that butyrate might decrease gp130 protein levels by attenuating gp130 protein stability. Through a series of experiments, we found that butyrate reduced the stability and accelerated the degradation of the gp130 protein by increasing its adaptor TRAF5 and enhancing their association, thus inhibiting the dimerization of gp130 and accelerating gp130 degradation.

Of note, in this study we found that enhancement of TRAF5 could accelerate the degradation of gp130, thereby reducing the level of gp130 in CRC cells and inhibiting activation of STAT3. The stability of gp130 was regulated by several kinds of post-translational modification. Ubiquitin-dependent proteasome degradation and caspase-induced proteolysis were reported to be the main pathways for gp130 degradation [46]. We speculated that increasement of TRAF5 may reduce gp130 stability by regulating its processing through the ubiquitin–proteasome pathway, and we would explore this issue in further study.

However, a study conducted by Yuan et al. revealed that butyrate inhibits the gene expression of gp80 and phosphorylation of STAT1 in the human CRC cell lines 228 and RKO. This research group also found that butyrate does not impact either the gene expression or protein level of gp130 [47]. These results are in contrast with ours, which may be due to the heterogeneity of cancer cells [48–50]. The expression of gp80 and gp130 differs in different kinds of cells. Therefore, the cellular response to butyrate can also differ. This possibility may be important for scientific research, but it was not explored in the present study. Further experiments are needed to explore this possibility.

In conclusion, we demonstrated that butyrate is a novel inhibitor of gp130 by targeting TRAF5. These results may contribute to further exploration of this metabolite in CRC.

## Conclusion

The fiber metabolite butyrate inhibits CRC development by reducing gp130 and inhibiting activation of IL-6/JAK2/STAT3 signaling. Furthermore, butyrate accelerates gp130 degradation by increasing its adaptor TRAF5. Due to these effects, butyrate is regarded as a novel therapeutic drug for CRC. However, specific mechanism of how butyrate regulates TRAF5 is unclear. Further researches and more human studies are still needed.

## Supplementary information

**Additional file 1: Table S1.** Specific primers for the qRT-PCR assay.

**Additional file 2: Figure S1.** The effects of 0.1 mM acetate, propionate, and valerate on HT-29 cell viabilities.

**Additional file 3: Figure S2.** NaB did not inhibit gene expression of gp80 and gp130. **a-b** The mRNA expression levels of gp80 and gp130 in cells treated with NaB (5 mM) for 24 h and IL-6 (25 ng/ml) for 30 min. (ns: not significant).

## Data Availability

The datasets used and/or analyzed during the current study are available from the corresponding author upon reasonable request.

## Abbreviations

CRC: Colorectal cancer
gp80: Glycoprotein 80
gp130: Glycoprotein 130
IL-6: Interleukin-6
JAK2: Janus tyrosine kinase 2
SCFAs: Short-chain fatty acids
NaB: Sodium butyrate
STAT3: Signal transducer and activator of transcription 3
TRAF5: Tumor necrosis factor receptor-associated factor 5
