# Supplementary material for: The fiber metabolite butyrate reduces gp130 by targeting TRAF5 in colorectal cancer cells
Source: Cancer Cell Int. 2020 Jun 3;20:212. doi: 10.1186/s12935-020-01305-9 (PMC7271451; doi:10.1186/s12935-020-01305-9)
Supplement: Supplementary file 1 — Additional file 1: Table S1. Specific primers for the qRT-PCR assay. [file 12935_2020_1305_MOESM1_ESM.docx]

**Additional file 2: Table S1. Specific primers for qRT-PCR assay.**

| **Gene** | **Forward Sequence (5’ – 3’)** | **Reverse Sequence (5’ – 3’)** |
| --- | --- | --- |
| GAPDH | TGCACCACCAACTGCTTAGC | GGCATGGACTGTGGTCATGAG |
| GP80 | CATTGCCCATGTTCTGAGGTTC | AGTAGTCTGTATTGCGGATGTC |
| GP130 | CGGACAGCTTGAACAGAATGT | ACCATCCCACTCACACCTCA |
