# Supplementary figures and images for: The fiber metabolite butyrate reduces gp130 by targeting TRAF5 in colorectal cancer cells
Source: Cancer Cell Int. 2020 Jun 3;20:212. doi: 10.1186/s12935-020-01305-9 (PMC7271451; doi:10.1186/s12935-020-01305-9)

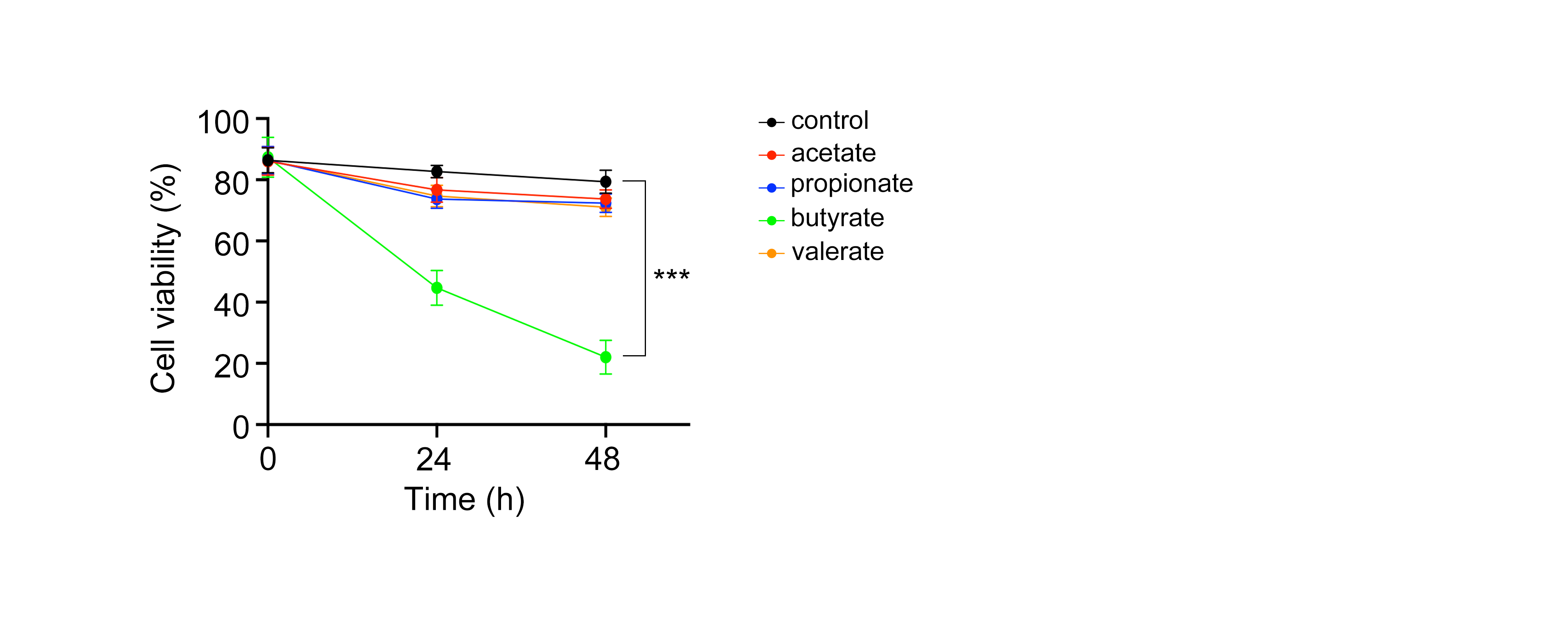

Supplement: Supplementary file 2 — Additional file 2: Figure S1. The effects of 0.1 mM acetate, propionate, and valerate on HT-29 cell viabilities. [file 12935_2020_1305_MOESM2_ESM.tif]

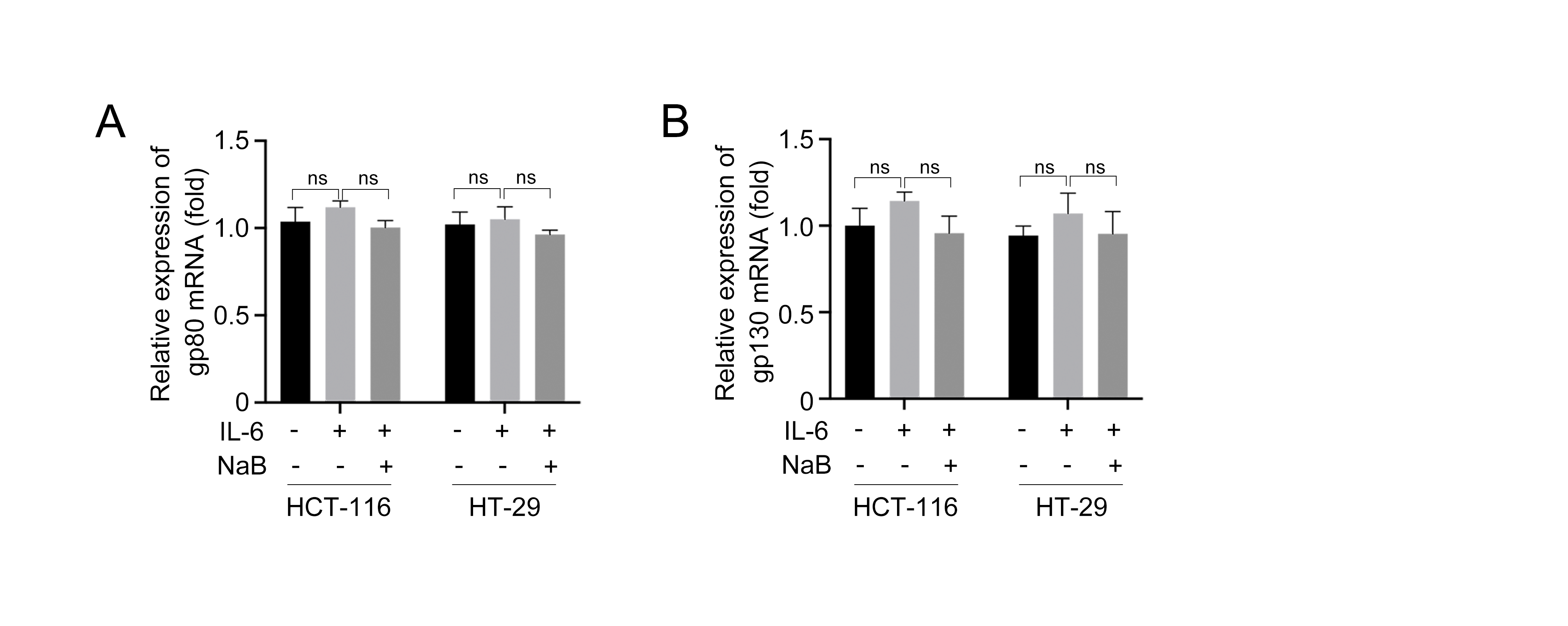

Supplement: Supplementary file 3 — Additional file 3: Figure S2. NaB did not inhibit gene expression of gp80 and gp130. a-b The mRNA expression levels of gp80 and gp130 in cells treated with NaB (5 mM) for 24 h and IL-6 (25 ng/ml) for 30 min. (ns: not significant). [file 12935_2020_1305_MOESM3_ESM.tif]
